# A simple and efficient gene functional analysis method for studying the growth and development of peach seedlings

**DOI:** 10.1093/hr/uhae155

**Published:** 2024-06-03

**Authors:** Jun Cheng, Yun Shao, Xinyue Hu, Liying Gao, Xianbo Zheng, Bin Tan, Xia Ye, Wei Wang, Haipeng Zhang, Xiaobei Wang, Xiaodong Lian, Zhiqian Li, Jiancan Feng, Langlang Zhang

## Abstract

Stable genetic transformation of peach [*Prunus persica* (L.) Batsch] still faces many technical challenges, and existing transient expression methods are limited by tissue type or developmental stage, making it difficult to conduct functional analysis of genes regulating shoot growth. To overcome this dilemma, we developed a three-step method for efficient analysis of gene functions during peach seedling growth and development. This method resulted in transformation frequencies ranging from 48 to 87%, depending on the gene. From transformation of germinating seeds to phenotyping of young saplings took just 1.5 months and can be carried out any time of year. To test the applicability of this method, the function of three tree architecture-related genes, namely *PpPDS*, *PpMAX4*, and *PpWEEP*, and two lateral root-related genes, *PpIAA14–1* and *−2*, were confirmed. Since functional redundancy can challenge gene functional analyses, tests were undertaken with the growth-repressor *DELLA*, which has three homologous genes, *PpDGYLA* (*DG*), *PpDELLA1* (*D1*), and *−2* (*D2*), in peach that are functionally redundant. Silencing using a triple-target vector (TRV2-*DG*-*D1*-*D2*) resulted in transgenic plants taller than those carrying just TRV2-*DG* or TRV2. Simultaneously silencing the three *DELLA* genes also attenuated the stature of two dwarf genotypes, ‘FHSXT’ and ‘HSX’, which normally accumulate DELLA proteins. Our study provides a method for the functional analysis of genes in peach and can be used for the study of root, stem, and leaf development. We believe this method can be replicated in other woody plants.

## Introduction

Peach is an economically important species in the Rosaceae family. Peach could be used as a model fruit tree in the Rosaceae family due to its relatively short juvenile period, a small genome size (approximately 230 Mb/haploid) and the availability of a high-quality genome sequence. However, functional gene analysis in peach lags behind other species of its family because there are no *in vitro* regeneration and transformation methods for generating transgenic peach plants. To the best of our knowledge, the generation of transgenic peach has been reported in four published articles, but most of these methods have proven to be impossible to repeat [1, 2]. Several methods of transient expression have been developed to analyse the functions of certain classes of genes, such as those related to volatile compound production, ABA biosynthesis, or anthocyanin production [3–6]. However, these methods have a narrow application scope. For example, the transience of the overexpression precludes observation of phenotypes that regulate organ growth and development. Recently, composite plants of peach were generated by infecting seedlings with *Agrobacterium rhizogenes* to induce transgenic adventitious hairy roots [7].

Virus-induced gene silencing (VIGS) is a powerful tool for functional gene analysis. VIGS induces RNA-mediated posttranscriptional gene silencing [8] and can be used for reverse genetics studies much like RNA interference (RNAi) and T-DNA insertional mutagenesis. As a tool, VIGS has many advantages over conventional techniques, such as ease of manipulation, high effectiveness and independence from stable transformation. VIGS technology has been successfully applied in many crops, including cotton [9], litchi [10], and pear [11].

The extensive application of VIGS may be attributed to its two outstanding merits: the systemic spread of gene silencing and long-term gene silencing. There are two ways that viruses or virus-induced siRNA spread throughout infected plants: cell-to-cell and/or long-distance through the phloem [12]. These mechanisms also function in VIGS experiments, such that photo-bleaching of young leaves can be seen far away from the infection sites when silencing *phytoene desaturase (PDS)* gene in tomato [13]. This systemic spread of gene silencing supports the VIGS-induced long-term silencing. Senthil-Kumar and Mysore [14] reported that VIGS was maintained for more than two years and could be transmitted to progeny seedlings in *Nicotiana benthamiana* and tomato. Bennypaul *et al.* [15] also reported that mosaic virus (BSMV)-based vector systems result in VIGS inheritance in wheat.

Peach tree architecture greatly impacts the productivity of orchards and has recently been attracting more attention as a breeding target [16]. Many traits related to peach tree architecture, such as branch angle, plant height and pendulous branch growth, were studied, and some key genes for these traits were identified [17–19] However, functional analysis of these key genes was conducted using heterologous expression. For instance, the functions of the *WEEP* gene, responsible for pendulous branch growth, and of the *GID1c* gene, responsible for plant height, were confirmed by silencing their homologous genes in plum [18, 19]. The function of *PpeTAC1*, which regulates branch angle, was analysed using the *attac1* mutant of Arabidopsis [17]. Up to now, there are no methods to support functional gene analysis in peach tree architecture.

**Figure 1:**
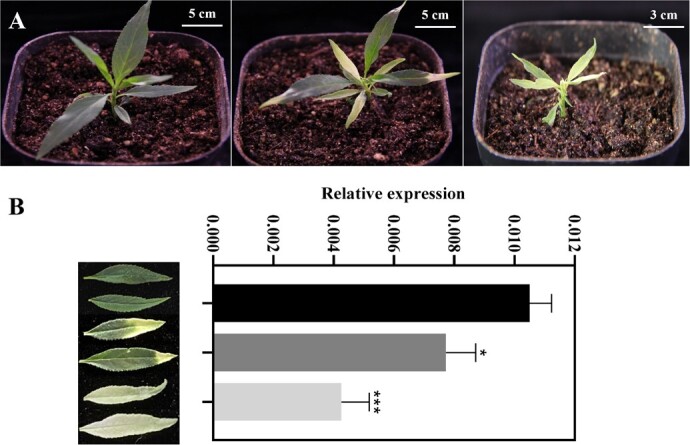
Silencing of *PpPDS* resulted in photo-bleached leaves in peach seedlings. **A** Seedlings infected with TRV2 (left) and TRV2-*PpPDS* (middle and right). The photos were taken 15 days post-infiltration. **B** Expression level of *PpPDS* in leaves exhibiting mild and severe bleaching. The values represent the average of three biological replicates The error bars indicate means ± standard deviation (SD) (*P < 0.05*;* ***P < 0.001)*.*

The tobacco rattle virus (TRV)-derived vector system has been applied in a wide range of species [20]. In this study, we employed a TRV-based vector to infect newly germinated peach seeds and successfully demonstrated the functions of genes related to tree architecture. Using this method, we efficiently confirmed the function of the *DELLA*, *DGYLA*, *MAX4*, and *WEEP* genes involved in plant height, branch outgrowth, and pendulous branch growth. In addition, *PpIAA14*, involved in lateral root formation, was cloned into pSAK277 and overexpressed by transformation of newly germinated peach seeds. We think that our reported method will be a powerful tool for analysing gene functions related to the growth and development of peach seedlings.

## Results

### Silencing of the peach *PDS* gene using TRV vector

The *PDS* gene encodes a phytoene desaturase enzyme and is commonly used to evaluate the silencing efficiency of endogenous gene. *PDS* silencing results in photo-bleaching of leaves by inhibiting carotenoid biosynthesis. To ensure that the TRV vector system could trigger virus-induced gene silencing in peach, the homologous gene of *PDS* (*PpPDS*) was identified from the peach genome and targeted for silencing. Three days post-infiltration (DPI), the remaining cotyledon of each peach seed infected with *Agrobacterium* carrying the TRV-*PpPDS* exhibited a photo-bleached phenotype (Fig. S1, see online supplementary material). By 15 DPI, 48.33% of TRV-*PpPDS* infected seedlings showed leaf photobleaching (Fig. 1A, Table 1). The leaves exhibiting mild and severe bleaching were collected for RT-qPCR analysis. The results showed that the completely white leaves had the lowest *PpPDS* transcript levels, while leaves exhibiting mild bleaching had a higher transcript level than the white leaves but a lower transcript level than control (Fig. 1B). These results demonstrated that the TRV vector system works in peach seedlings.

**Table 1:** Frequency of seedlings with expected phenotype

Gene	No. of infected seeds	No. of plants with expected phenotype	Frequency / %
*PpPDS*	36/36/45	15/18/24	48.33 ± 5.69
*PpMAX4*	36/28/28	30/24/26	87.33 ± 5.13
*PpWEEP*	45/35/40	24/19/24	55.80 ± 3.60
*PpDGYLA*	85/20/20	44/10/9	49.00 ± 3.61
*PpDGYLA-DELLA1-DELLA2*	75/20/20	44/10/10	53.00 ± 5.20
*PpIAA14–1*	30/30/40	20/19/25	64.17 ± 2.47
*PpIAA14–2*	35/34/38	24/18/22	60.00 ± 8.19

Frequency (%) = (Number of plants showing expected phenotype/Total number of infected plants)^*^100%. Three biological replicates were conducted.

### Silencing of *PpMAX4* affects branch outgrowth and root system of peach seedlings

**Figure 2:**
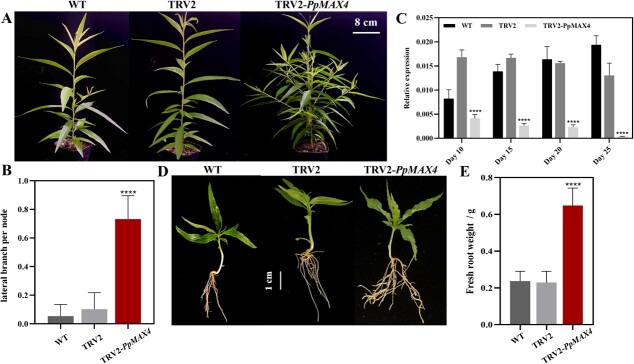
Silencing of *PpMAX4* increased the number of lateral branches and lateral roots. **A** Lateral branching in wild-type seedlings (left) and seedlings infected with TRV2 (middle) and TRV2-*PpMAX4* (right). The photos were taken 28 days post-infiltration (DPI). **B** Analyse the number of lateral branches at each node of the peach seedling 28 DPI (by comparing the branch number to the internode number). **C** Expression levels of *PpMAX4* in shoot tips collected 10, 15, 20, and 25 DPI. The values represent the average of three biological replicates (^****^P < 0.0001). **D** Lateral roots of seedlings 10 DPI. **E** Fresh roots weight of seedlings 10 DPI. **B** and **E** Error bars indicate means ± standard deviation (SD) from three biological replicates. ^****^ indicates significant differences at *P* < 0.0001 between TRV2 and TRV2-*PpMAX4*.

Strigolactones play a negative role in branch outgrowth. Downregulation of *MAX4*, a key gene in strigolactone biosynthesis, increases the number of lateral branches and adventitious roots in poplar [21]. In this study, only one homologous gene of *MAX4* (*PpMAX4*) was identified in the peach genome, with a similar gene structure compared to *AtMAX4* in terms of exons and introns distribution (Fig. S2, see online supplementary material). By 14 DPI, the seedlings infected with TRV2 had no lateral branches, while nine TRV2-*PpMAX4* seedlings showed growth of almost all lateral buds (Fig. S3, see online supplementary material). The average number of lateral branches per node were calculated 25 DPI with TRV2 or TRV2-*PpMAX4*. Peach seedlings infected with TRV2-*PpMAX4* had an average of 0.8 lateral branches per node, while the uninfected and TRV2 plants had an average of 0.05 lateral branches per node (Fig. 2A and B). The frequency of *PpMAX4* gene silencing reached 87.33% (Table 1). Shoot tips were collected 10, 15, 20, and 25 DPI for RT-qPCR analysis. Compared with the controls, the transcript levels of *PpMAX4* were significantly downregulated in peach seedlings infected with TRV2-*PpMAX4* at all days analysed (Fig. 2C). The *PpMAX4* silencing was stable for 25 days as the seedling grew. In addition, *PpMAX4*-silenced plants (10 days old) had more root fresh weight compared to the TRV2 control (Fig. 2D and E). These results demonstrated that silencing of *PpMAX4* increases the lateral number and root fresh weight of peach seedlings.

### Silencing of *PpWEEP* results in a pendulous growth of peach seedlings

**Figure 3:**
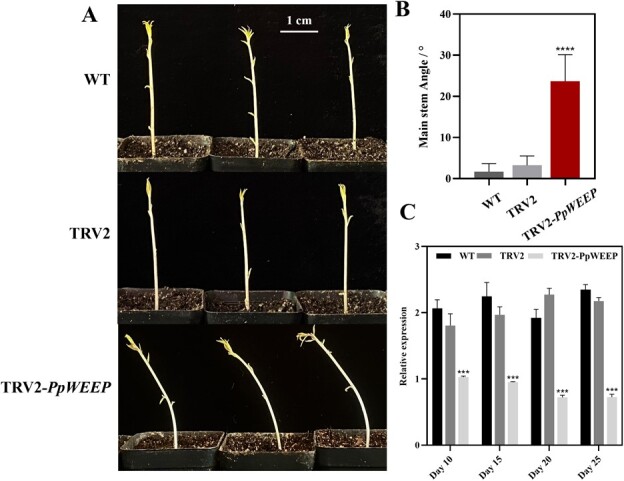
Silencing of *PpWEEP* resulted in a phenotype of pendulous branch growth in peach seedlings. **A** Upright and pendulous growth of dark-grown seedlings. Uninfected wild-type seeds and seeds inoculated with *Agrobacterium* harboring the empty TRV2 vector and TRV2-*PpWEEP* were cultured in pots under dark conditions. The photos were taken at 15DPI. **B** Measure of the angle of the main stem to the vertical. Error bars indicate means ± standard deviation (SD) from three biological replicates. ^****^ indicate significant differences at *P* < 0.0001 between TRV2 and TRV2-*PpWEEP.***C** Expression level of *PpWEEP* in shoot tips collected at 10, 15, 20, and 25 days post infiltration. The values represent the average of three biological replicates (^*^P < 0.05; ^***^P < 0.0001).

A pendulous or weeping architecture of peach branches is caused by a loss-of-function mutation in *PpWEEP* [19], which seems to alter normal gravitropic responses. In this study, silencing of the *PpWEEP* gene resulted in a pendulous growth phenotype in peach seedlings grown in the dark, but not under light (Fig. 3A). The dark-dependent phenotype may occur because phototropism of shoot tips is strong enough to mitigate the loss of *PpWEEP* function in the light. The angle of the main stem to the vertical was analysed in seedlings grown in the dark. Peach seedlings infected with TRV2-*PpWEEP* had an average angle of 24°, while the uninfected and TRV2 plants had an average angle of 3° (Fig. 3B). The frequency of *PpWEEP* gene silencing was 55.8% (Table 1). At 10, 15, 20, and 25 DPI, shoot tips were collected for RT-qPCR analysis. Compared with the WT and TRV2 controls, *PpWEEP* transcript levels were significantly downregulated in peach seedlings infected with TRV2-*PpWEEP* (Fig. 3C). Silencing also confirmed that *PpWEEP* plays a key role in upright tree architecture, while its loss results in pendulous branch growth in peach.

### Silencing of *PpDGYLA*, *PpDELLA1*, and *−2* increases the height of peach seedlings

**Figure 4:**
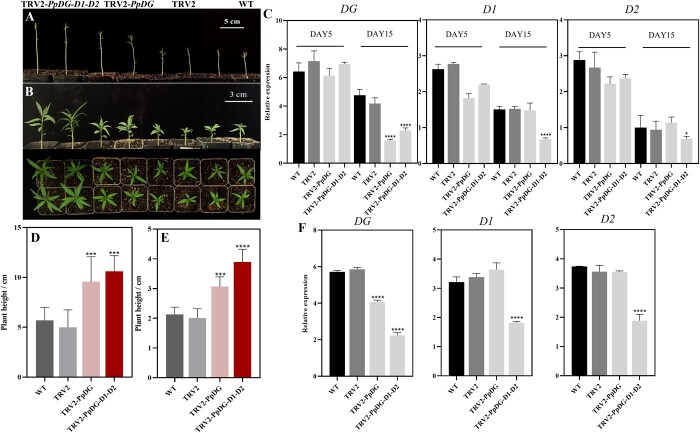
Silencing of *DELLA* genes increased plant height. Seedlings were cultured in the dark (**A**) or treated with PBZ (**B**). The photos were taken 10 DPI (**A**) and 15DPI (**B**). **D** and **E** Analysis of plant height of dark-grown (**D**) and PBZ-treated (**E**) seedlings. **C** and **F** Expression levels of *PpDG*, *PpD1*, and *PpD2* in shoot tips collected 5, 15 days (C, dark-grown), and 25 days (F, PBZ-treated) post-infiltration (^*^P < 0.05; ^****^P < 0.0001). The values represent the average of three biological replicates. **D** and **E** Error bars indicate means ± standard deviation (SD) from three biological replicates. ^***^ and ^****^ indicate significant difference at *P* < 0.001 and *P* < 0.0001 between TRV2 and TRV2-*PpDGYLA* or TRV2*-DG-D1-D2.*

DELLA proteins are well-known repressors of GA signaling. Our previous work suggested that peach has three homologues of *DELLA*, namely *PpDGYLA* (*DG*), *PpDELLA1* (*D1*) and *−2* (*D 2*), which play negative roles in internode elongation of peach [22, 23]. In view of the functional redundancy among the three peach *DELLA* genes, three fragments from the coding sequences of *DG*, *D1*, and *D2* were amplified and then simultaneously inserted into a TRV2 vector (Fig. S4, see online supplementary material). Peach seedlings are different in genetic material and show to some extent variation in plant height, which raise the bar to analyse the change of plant height induced by TRV2-*DG* or TRV2-*DG*-*D1*-*D2*. In this study, there was no significant discrepancy in plant height among these seedlings grown from seeds infected with TRV2, TRV2-*DG*, and TRV2-*DG*-*D1*-*D2*. To reduce the natural variation in plant height, we designed two experiments: peach seedlings were cultured in the dark or were grown in the light and treated with paclobutrazol (PBZ, a GA biosynthesis inhibitor). Both of these treatments efficiently reduced the variation of plant height in wild-type peach seedlings (Fig. S5, see online supplementary material). The plant height of dark-grown seedlings from seeds inoculated with *Agrobacterium* carrying the silencing vectors were analysed 10 DPI. In the dark, the TRV2-*DG*-*D1*-*D2* plants were taller than the TRV2-*DG*, and the TRV2-*DG* plants were taller than uninoculated control (Fig. 4A and D). RT-qPCR showed that the three peach *DELLA* genes were downregulated in the TRV2-*DG*-*D1*-*D2* plants, while only *PpDGYLA* was downregulated in the TRV2-*DG* plants (Fig. 4C). Similar results were observed when peach seedlings were treated with 300 mg/L PBZ (Fig. 4B, E, and F).

### Silencing of the DELLA genes disrupts genetically dwarfed peach seedlings

**Figure 5:**
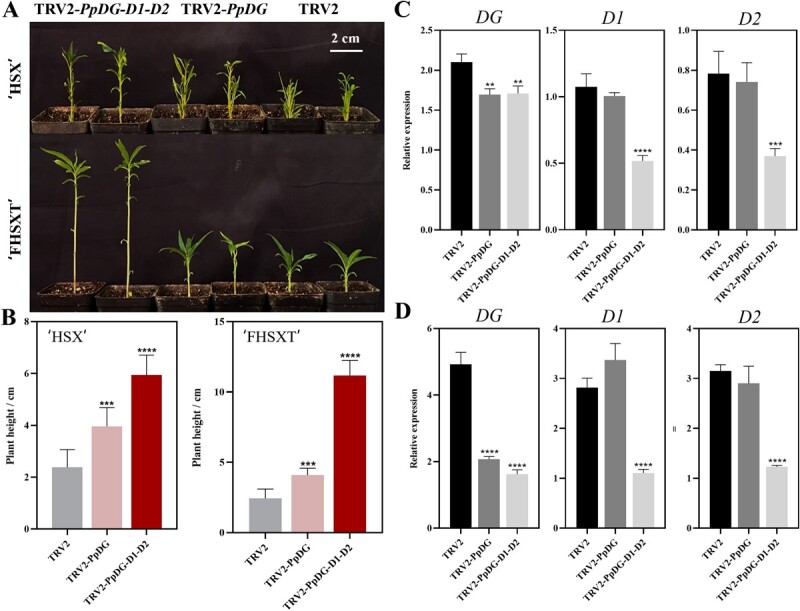
The dwarf phenotypes of ‘FHSXT’ and ‘HSX’ were attenuated by silencing of peach *DELLA* genes. **A** The plant height of seedlings. Photos were taken 15 DPI. **B** Analysis of plant height of the ‘FHSXT’ and ‘HSX’ dwarf genotypes. Error bars indicate means ± standard deviation (SD) from three biological replicates. ^***^ and ^****^ indicate significant differences at *P* < 0.001 and *P* < 0.0001 between TRV2 and TRV2-*PpDGYLA* or *TRV2-DG-D1-D2.* Expression levels of *PpDGYLA*, *PpDELLA1*, and *PpDELLA2* in shoot tips from ‘HSX’ (**C**) and ‘FHSXT’ (**D**) collected 25 DPI. The values represent the average of three biological replicates (^**^P < 0.01; ^***^P < 0.001; ^****^P < 0.0001).

Our previous work demonstrated that a mutation in the gibberellic acid receptor *PpGID1c* causes a GA-insensitive dwarf phenotype in peach through its increased accumulating of DELLA protein [23]. To confirm this conclusion, peach pits collected from two dwarf genotypes, ‘HongShouXing (HSX)’ and ‘FengHuaShouXingTao (FHSXT)’ were used for VIGS assay. Plant height was analysed 15 DPI. In the two dwarfed backgrounds, TRV2-*DG*-*D1*-*D2* plants were taller than the TRV2-*DG* plants, and the TRV2-*DG* plants were taller than the genotype controls. The change was most drastic in ‘FHSXT’, with the plant height of TRV2-*DG*-*D1*-*D2* plants extremely higher than the TRV2-*DG* and TRV2 control plants (Fig. 5A and B). RT-qPCR showed that the three peach *DELLA* genes were downregulated in dwarf peach seedlings infected with TRV2-*DG*-*D1*-*D2*, while only *PpDGYLA* was downregulated in the TRV2-*DG* plants (Fig. 5C and D). Together, these results demonstrated that the TRV vector system could silence *DELLA* genes, resulting in increased plant height of peach seedlings.

### Overexpression of *PpIAA14–1* and *PpIAA14–2* mutants affected lateral root development in peach

**Figure 6:**
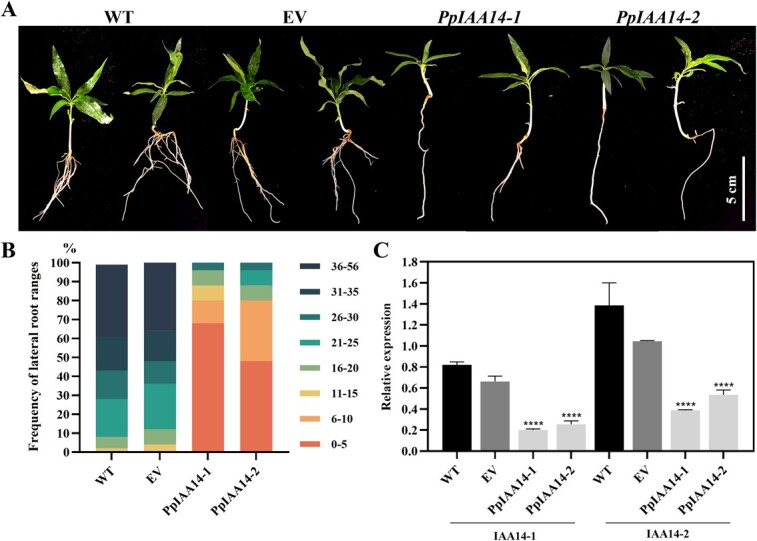
Overexpressing *PpIAA14–1^P102S^* and *PpIAA14–2^P108S^* inhibited the number of lateral roots in peach. **A** Whole seedlings removed from soil 14 days post-infection. **B** Analysis of lateral root numbers at 14 DPI. In these seedlings, the minimum number of lateral roots was 0, while the maximum was 56. The frequency for each range indicated by color are shown. **C** Transcript levels of *IAA14–1* and *IAA14–2* were analysed in the main root 10 days post-infection. The values represent the average of three biological replicates. (^****^P < 0.0001) (EV represent pSAK277-*GFP*).

Previous work demonstrated that a gain-of-function mutation in the *IAA14* gene stops lateral root formation in Arabidopsis [24]. In this study, two homologous genes, *PpIAA14–1* and *PpIAA14–2*, were identified from the peach genome. According to Fukaki *et al.* [24], a single nucleotide substitution was introduced into *PpIAA14–1* and *PpIAA14–2*, which converted the proline at position 102 (PpIAA14–1) or 108 (PpIAA14–2) into a serine (Fig. S6, see online supplementary material). To boost the frequency of transient expression, removal of one cotyledon was tested using the common *Agrobacterium* strain GV3101. Our result showed that removal of one cotyledon significantly improved transformation efficiency (Fig. S7, see online supplementary material). Then, *35S-PpIAA14–1^P102S^-GFP* and *35S-PpIAA14–2^P108S^-GFP* were delivered into the germinated seeds. At four DPI, the lateral root phenotypes were analysed. Compared to the *GFP* control, the number of lateral roots significantly decreased in peach seedlings expressing *PpIAA14–1^P102S^-GFP* or *PpIAA14–2^P108S^-GFP* (Fig. 6A). Among these 200 seedlings infected with *PpIAA14–1^P102S^-GFP* and *PpIAA14–2^P108S^-GFP*, there are 160 seedlings having a lateral root number ranging from 0 to 10. One hundred seedlings were infected by *Agrobacterium tumefaciens* containing *35S*-*GFP* and 88 seedlings had 21–56 lateral roots (Fig. 6B). Analysis of the transcript levels of *PpIAA14–1^P102S^-GFP* and *PpIAA14–2^P108S^-GFP* using RT-qPCR showed that *GFP* was overexpressed in roots expressing *PpIAA14–1^P102S^-GFP* and *PpIAA14–2^P108S^-GFP*, but the transcript levels of *PpIAA14–1* or *PpIAA14–2* were downregulated (Fig. 6C). These results demonstrated that overexpression of *PpIAA14–1^P102S^* or *PpIAA14–2^P108S^* could significantly reduce lateral root formation in peach seedlings.

### Discussion

Peach is one economically important species that has long proven recalcitrant to *in vitro* regeneration and genetic transformation [2, 7]. Existing transient expression methods have a narrow range of application. Therefore, the development of a method to support functional gene analysis in peach is of paramount importance. The TRV vector system is widely used to silence endogenous genes in many plant species. The TRV vector system has been used to analyse genes regulating fruit firmness in peach through transient fruit transformation [25]. In this study, our results showed that six genes (*PpPDS*, *PpMAX4*, *PpDGYLA*, *PpDELLA1*, *−2*, and *PpWEEP*) were efficiently silenced for a relatively long time (25 days in the TRV2-*PpMAX4* assay) in peach seedlings, with effects in multiple vegetative tissues as the seedlings developed. Compared to existing transient expression methods [3–6], long-term gene silencing could be achieved with this method, which is essential for analysing genes involved in shoot development. Xu *et al.* [7] and Wu *et al.* [26] reported a method by which transgenic adventitious hairy roots could be obtained. Compared with the method we developed, this needs a procedure of tissue culture and restricted to the application on root. Together, our study provides a simple and effective tool for functional gene analysis in peach.

Gene silencing efficiency is a crucial indicator that determines whether the TRV vector system is suitable for its use in peach. In this study, we analysed genes regulating tree architecture-related traits (plant height, branch number, and lateral root number). These traits generally belong to quantitative character. The expected change induced by silencing *PpMAX4*, *PpWEEP, PpDGYLA*, *PpDELLA1*, and *PpDELLA2* is not a clear qualitative change. To enhance the reliability of the results, several ways were used to reduce variation of the observed traits. For peach seedlings within 30 d age, almost no branches outgrow. Therefore, lateral branches in *PpMAX4* silencing assay were calculated 25 DPI. To analyse the gene function of *PpDGYLA*, *PpDELLA1*, and *−2*, we reduced the range of plant height variation by using the seedlings cultured in dark conditions or treated with PBZ. In addition, to overcome the challenge posed by functional redundancy, the TRV2-*DG-D1-D2* vector was constructed and the three *DELLA* genes were silenced simultaneously. This result is similar to the use of the TRV vector system to efficiently knockdown 12 members of the highly homologous *E2* gene family [27]. By these methods, assays obtained a relatively high gene silencing frequency in peach, ranging from 48.33% to 87.33%.

TRV-induced gene silencing is strongly affected by many factors [28]. Our results showed that genetic background is another factor affecting gene silencing frequency of the TRV vector system. We collected peach pits from two dwarf peach genotypes ‘HSX’ and ‘FHSXT’. In the TRV-*DG* and TRV2-*DG*-*D1*-*D2* assay, we found that the frequency of gene silencing was significantly higher in ‘FHSXT’ than in ‘HSX’. Peach is both self-pollinated and cross-pollinated. This means that seeds from the same plant may carry genetic differences, leading to greater variation in growth and development of seedlings, which can threaten functional gene analysis efforts. However, we also believe that using peach seedlings increases the likelihood of identifying genes that play key roles in the development of specific traits. The influence of a single gene on plant phenotype is complex and changes in different genetic backgrounds. The phenotypes of a mutant allele are not a simple result of a single gene mutation, but are instead due to interactions of the allele with other genes and the environment [29]. Therefore, a gene with a powerful function in most genetic backgrounds is most likely to play a key role in a specific trait.

**Figure 7:**
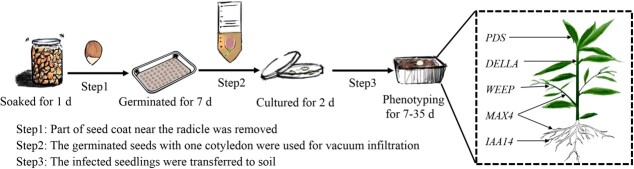
A simple procedure for transformation of peach seedlings.

In conclusion, we provide a simple pipeline schematic for our method (Fig. 7). In Step 1, part of the seed coat and one cotyledon are removed from an imbibed seed. In Step 2, the germinated seeds with one cotyledon are inoculated with *Agrobacterium* using vacuum infiltration. In Step 3, the infected seedlings were transferred into soil for phenotyping. This transformation method had three distinct advantages, especially for TRV-induced gene silencing in peach seedlings. Firstly, the method is highly efficient. Our results showed a greater than 48.33% gene silencing frequency that could be accomplished within 1.5 months and carried out at any time of year. Secondly, gene functions related to growth and development of the shoot, root, and leaf can be analysed using this method. Thirdly, this method can likely be applied to other woody plants, such as plum and almond. However, we do wish to pursue greater transformation frequency and to further study the duration, stability, and heritability of the gene silencing achieved with this method.

## Materials and methods

### Plant material and growth conditions

Peach pits were collected from the wild peach Maotao and two dwarf genotypes, ‘HSX’ and ‘FHSXT’, which are maintained at the Fruit Tree Germplasm Repository of Henan Agricultural University (Henan Province, China). ‘HSX’ and ‘FHSXT’ are two dwarfing varieties in peach. Their dwarfing traits are caused by the accumulation of DELLA proteins. These peach pits were stored at 4°C for more than 60 days before seeds were taken from the pit and immersed in distilled water for 1 day. A portion of the seed coat near the radicle was removed, and then the seeds were placed on wet filter paper for 7 days at 24 ± 1°C in the dark. During germination, the radicle would elongate, and germinated seeds with a radicle of 1 ± 0.2 cm in length were selected for transformation.

### Vector construction

The tobacco rattle virus (TRV)-derived vectors pTRV1 and pTRV2 used in gene silencing assays were described in [30]. Portions of the coding sequences of *PpPDS* (1–167 bp), *PpMAX4* (1236–1500 bp), *PpWEEP* (185–387 bp), and *PpDGYLA* (150–300 bp) were amplified and inserted into the vector pTRV2. Three fragments cloned from *PpDGYLA* (150–300 bp), *PpDELLA1* (150–300 bp), and *PpDELLA2* (150–300 bp) were amplified and connected into a fragment, named *DG*-*D1*-*D2*, which was then inserted into the vector pTRV2. The primers used to amplify the fragments are listed in Table S1 (see online supplementary material) along with each Gene ID.

The binary vector pSAK277, containing a *35S*-*GFP* cassette, was used for gene overexpression assays. The coding sequences of *PpIAA14–1* and *PpIAA14–2* were cloned. According to a previous study [24], overlapping PCR was used to convert the proline at amino acid (aa) position 102 in PpIAA14–1 and at position 108 in PpIAA14–2 to serine. The primers used in the overlapping PCR assay are listed in Table S1 (see online supplementary material). After mutation, the pSAK277-*PpIAA14–1^P102S^-GFP* and pSAK277-*PpIAA14–2^P108S^-GFP* vectors were constructed. cDNA from the peach cultivar ‘QiuMiHong’ was used as the template for the PCRs for subsequent cloning.

### Vacuum infiltration

The prepared vectors were delivered into *A. tumefaciens* GV3101, which then were cultured at 28°C on LB plates containing 50 mg/L kanamycin or spectinomycin. Positive clones were selected and cultured in 400 μL LB liquid medium (containing 50 mg/L kanamycin or spectinomycin) overnight. Then, 100 μL of the *A. tumefaciens* cultures were added to 100 mL LB medium (containing 50 mg/L kanamycin or spectinomycin) and incubated at 28°C with shaking at 180 rpm for 12 h.

The *A. tumefaciens* culture was centrifuged at 5000 rpm to collect agrobacterium cells, which were then suspended in MES suspension buffer (100 μM acetosyringone, 10 mM MgCl_2_, 10 mM MES; pH 5.6). The concentration was adjusted to OD_600nm_ = 0.7–0.9. Then, the *A. tumefaciens* suspense was incubated at room temperature for 3 hours.

After germination on filter paper for 7 days, seeds with a radicle of 1 ± 0.2 cm in length were selected and then the rest of the seed coat and one cotyledon were removed from the germinated seeds. Seeds with greenish plantule were then immersed in the prepared *A. tumefaciens* suspension and placed under vacuum (−0.8 Mpa) for 20 min. After vacuum infiltration, seeds were rinsed in sterile water, cultured on filter paper for two more additional days, then transferred into soil. In TRV2-*PpPDS*, TRV2-*PpMAX4*, and pSAK277-*PpIAA14–1^P102S^*/*PpIAA14–2^P108S^* assays, seedlings were cultured at room temperature and normal light conditions. In TRV2-*PpWEEP*, seedlings were cultured at room temperature and dark conditions. In TRV2-*PpDG* and TRV2-*DG-D1-D2* assay, seedlings were cultured at light or dark conditions.

### RT-qPCR **analysis**

In TRV2-*PpMAX4*, TRV2-*PpWEEP*, TRV2-*PpDG*, and TRV2-*DG-D1-D2* assay, shoot tips were collected to analyse transcript level. In TRV2-*PpPDS* assay, to confirm the relationship between phenotype and gene expression level, whole leaves exhibiting mild and severe bleaching were collected, respectively. In pSAK277-*PpIAA14–1^P102S^*/*PpIAA14–2^P108S^* assay, root tip was collected to analyse transcript level. Samples of each technical replicates were prepared using more than three plants. Three biological replicates were conducted in each assay of RT-qPCR analysis. RNA was prepared and first-strand cDNA synthesis was carried out using the Total RNA Rapid Extraction Kit (Huayueyang Biotechnology, Beijing, China) and HiScript III RT SuperMix for RT-qPCR (+gDNA wiper) (Vazyme, Nanjing, China). A 20-μL reaction for quantitative RT-qPCR contained 1 × ROX reference dye, 10 μL of 2 × SYBR premix Ex Taq II (TaKaRa), 0.4 μM of each primer, and 100 ng of template cDNA. The peach *EF2* gene (Prupe.4G138900) was used as the reference for normalization [31]. The primers used for RT-qPCR are listed in Table S1 (see online supplementary material).

### Scoring the phenotypes of TRV-induced gene silencing and *PpIAA14* overexpression

The plants following TRV-induced gene silencing were scored according to wild-type and expected phenotypes. The frequency of gene silencing was determined according to Senthil-Kumar and Mysore [14]. The frequency of gene silencing was calculated by comparing the number of plants with an expected phenotype to the total number of plants infected with TRV constructs. The phenotype and frequency of *PpPDS* gene silencing were determined according to Senthil-Kumar and Mysore [14]. The expected phenotypes of *PpMAX4, PpWEEP*, and *PpDELLA* were different, as described below. For *PpMAX4*, the frequency of gene silencing was calculated 25 days after infiltration. The expected phenotype of *PpMAX4*-silencing was a seedling with a greater number of lateral branches than the average branch number of WT and TRV2 (0.05 branches/a node). For *PpWEEP*, the frequency of gene silencing was calculated 15 days after infiltration. The expected phenotype of *PpWEEP*-silencing was seedlings with an angle larger than the average angle of WT and TRV2 (3°). For *PpDELLA*, the frequency of gene silencing was calculated 15 days after infiltration. The expected phenotype of silencing the three *PpDELLA* genes was seedlings with a plant height higher than the average plant height of WT and TRV2 (seedling grown in the dark, 5.7 cm; in the light, 2.1 cm; and seedlings of dwarf peach genotype, 2.4 cm).

The expected phenotype of the plants inoculated with pSAK277-*PpIAA14–1^P102S^-GFP* and pSAK277-*PpIAA14–2^P108S^-GFP* was seedlings with a lower average number of lateral roots compared with WT and *GFP* control seedlings (32.75 and 31.92).

### Statistical analysis

The Statistical Product and Service Solutions (SPSS) software (IBM Co., Armonk, NY, USA) was used to conduct the statistical analyses. All experimental data were tested by Student’s *t*-test. Prism 8.0.1 software (GraphPad, San Diego, CA, USA) was used to generate figures.

## Supplementary Material

Web_Material_uhae155

## Data Availability

All relevant data are included in the article and its supporting materials.
