# Supplementary material for: A simple and efficient gene functional analysis method for studying the growth and development of peach seedlings
Source: Hortic Res. 2024 Jun 3;11(7):uhae155. doi: 10.1093/hr/uhae155 (PMC11246241; doi:10.1093/hr/uhae155)
Supplement: Web_Material_uhae155 [file web_material_uhae155.zip › supplymental material.docx]

**Table S1** **List of primers used in this study.**

| **Primer** |  | **sequence** | |
| --- | --- | --- | --- |
| **Primers for vector construction**   \| *TRV2-PpMAX4*（Prupe.1G448400） \| 5’- ATTCTGTGAGTAAGGTTACC**GAATTC**CTACAACTTCCGCCACCTCT -3’  5’- TCTTCGGGACATGCCCGGGC**CTCGAG**CCATCTGCGAGCATAACCA -3’ \| \| --- \| --- \| \| *TRV2-PpPDS*（Prupe.1G174100） \| 5’-ATTCTGTGAGTAAGGTTACC**GAATTC**ATGTCTCAGTGGGCTTGTGTCT-3’  5’-TCTTCGGGACATGCCCGGGC**CTCGAG**CTTCCATAAATAGTTTGTGGGCTT-3’ \| \| *TRV2-PpWEEP*（Prupe.3G200700） \| 5’-TGTGAGTAAGGTTACC**GAATTC**TCGGTTTATAAGACGGTGCC-3’  5’-TCTTCGGGACATGCCCGGGC**CTCGAG**TGGTTCCAGCTTCAAGGAAA-3’ \| \| *TRV2- PpDGYLA*（Prupe.7G236100） \| 5’-TGTGAGTAAGGTTACC**GAATTC**GCTCAGCGCCTCGAGC-3’  5’-TCTTCGGGACATGCCCGGGC**CTCGAG**GATAAGGGGCTGGTGGT-3’ \| \| *TRV2- PpDGYLA- PpDELLA1*（Prupe.3G162500）- *PpDELLA2*（Prupe.1G329600） \| 5’-TGTGAGTAAGGTTACC**GAATTC**GCTCAGCGCCTCGAGC-3’  5’-CGGCAAGGATAAGGGGC-3’  5’-AGCCCCTTATCCTTGCCG-3’/ 5’-CAACACGGCGGAGAGATCA-3’  5’-TCTGATCTCTCCGCCGTGTT-3’  5’-TCTTCGGGACATGCCCGGGC**CTCGAG**CGATAGATCCGACGGGTTATAG-3’ \| \| *PpIAA14-1*（Prupe.1G027600） \| 5’- GTGGATCCAAA**GAATTC**ATGGCAGCATCTGCTGTTAGC -3’  5’- GATCGCACTG**A**TGGCCAAC -3’  5’- GTTGGCCA**T**CAGTGCGATC -3’  5’- CTCCTTTACCCAT**GAATTC**GCTCCTGTTTTTGCATTTCTCC -3’ \| \| *PpIAA14-2*（Prupe.6G343800） \| 5’- GTGGATCCAAA**GAATTC**ATGAGTACCGTGACTCATGAAG -3’  5’- AAGATCGGACGG**A**TGGCCAA -3’  5’- TTGGCCA**T**CCGTCCGATCTT -3’  5’- CTCCTTTACCCAT**GAATTC**ACATCTGTTCTTGCACTTCTCCACG -3’ \| | | | |
| **Primers for qPCR**   \| *EF2* \| 5’-AGCAGGCTCTTGGTGGTATCT-3’/5’-GATTCAATGACGGGGAGGTAG-3’ \| \| --- \| --- \| \| *PpGFP* \| 5’-CAACAGGATCGAGCTTAAGGG-3’/5’-GCTTCCATCTTCAATGTTGTGTC-3’ \| \| *PpIAA14-1* \| 5’-AAGGATGGCGATTGGATGCT-3’/5’-CTCCATAGCCCTTGGAGCAA-3’ \| \| *PpIAA14-2*  *PpMAX4*  *PpPDS*  *PpWEEP*  *PpDGYLA*  *PpDELLA1*  *PpDELLA2* \| 5’-GGACGAGGGTGAAAAGAGCA-3’/5’-CATTCCATCTGACCCGCAGT-3’  5’-TGCTGGTGATTGTGCATTTG-3’/5’-CAGCAACAAATACTAAAGGGCA-3’  5’-GGTGAAAACACCAAGGTCGG-3’/ 5’-AATTCCGGTGTCCTGATGGAA-3’  5’-TGCCACATGAAATGGGGAGA-3’/ 5’-CTGAGAGGCAAGATGGAGCC-3’  5’-TTAGGGCTCTGCATTTGGGTT-3’/5’-GGTTGGCTGTGGTTGCTTCA-3’  5’-*GGATGAGTTGCTTGCCGTTT*-3’*/* 5’-*AGCAGAGCCCATGACCATCT*-3’  5’-GCAGGTGCTATGCGAAAAGTT-3’/ 5’-CGGAGAATGAGTGGTCGATTG-3’ \| | | | |

| **P**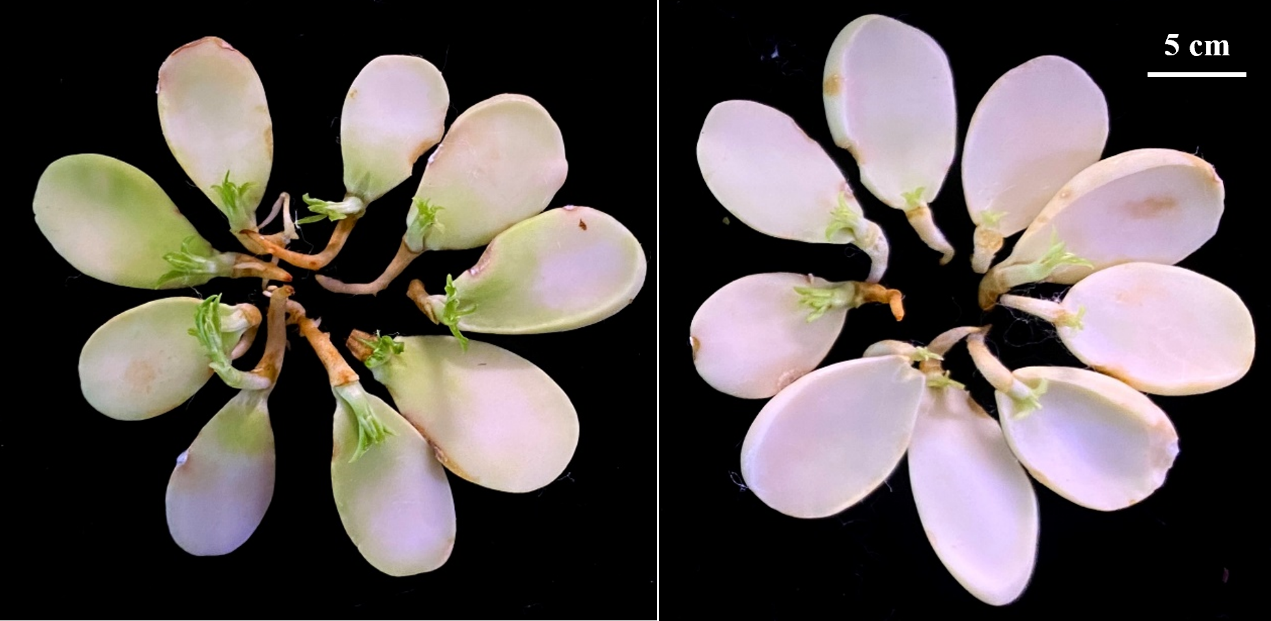  **Figure S1** The germinated seeds were infected with TRV2 (left) and TRV2-*PpPDS* (right). The photos were taken at 3 days post infiltration, and a photo-bleached phenotype of cotyledon were observed.  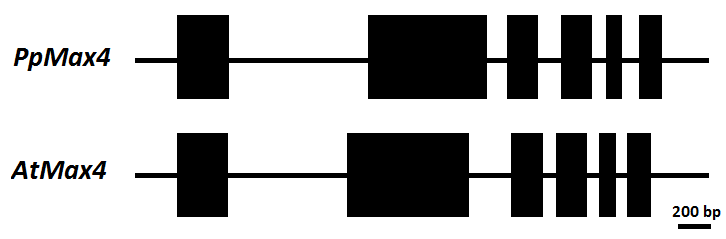  **Figure S2** A similar gene structure of *MAX4* between peach and Arabidopsis. Black box = exon.  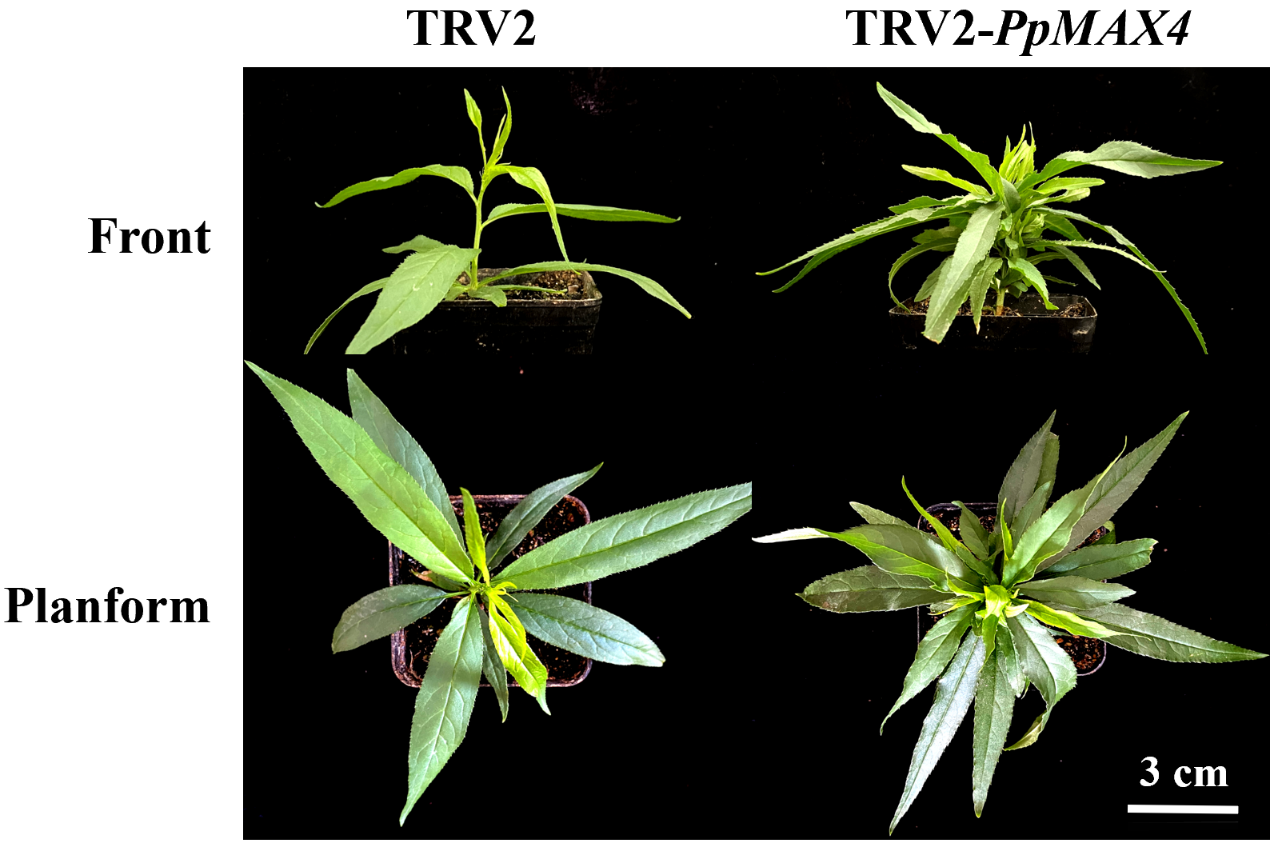  **Figure S3** The phenotype of lateral branch of seedlings at 14 day post infiltration  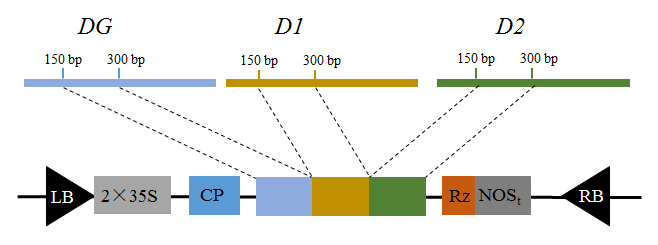  **Figure S4** The construction of TRV2-*DG*-*D1*-*D2* vector.  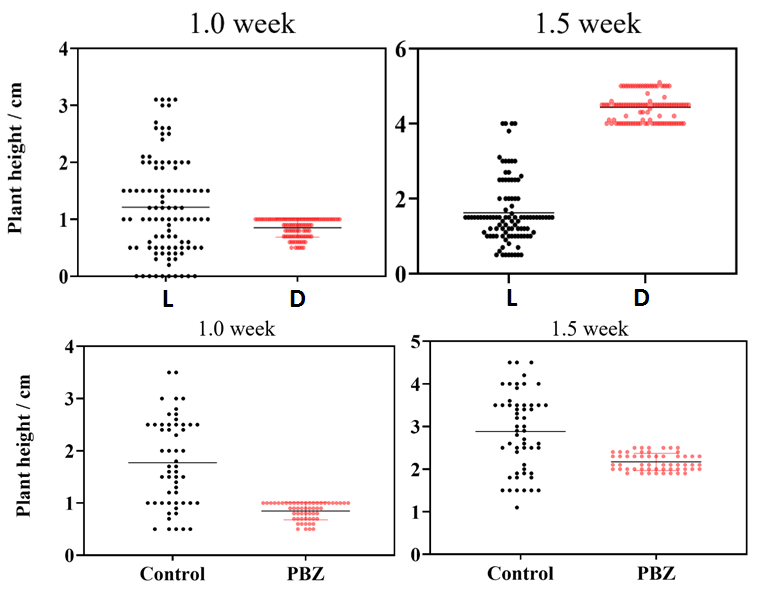  **Figure S5** Variation in plant height of peach seedlings were cultured under light (L) and dark (D) condition (top) and ariation in plant height of peach seedlings were treated with PBZ and control (Down)  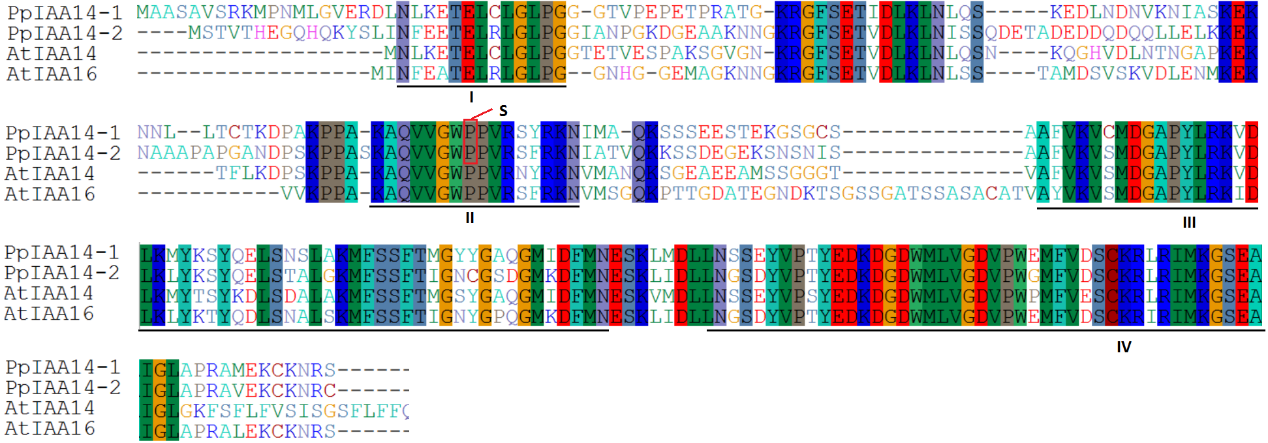  **Figure S6** Multiple sequence alignment showing the four conserved domains (I-IV) in PpIAA14-1 and PpIAA14-2. The conversion of the amino acid at position 102 (PpIAA14-1) or 108 (PpIAA14-2) from a proline (P) to a serine (S) are highlighted using a red box.  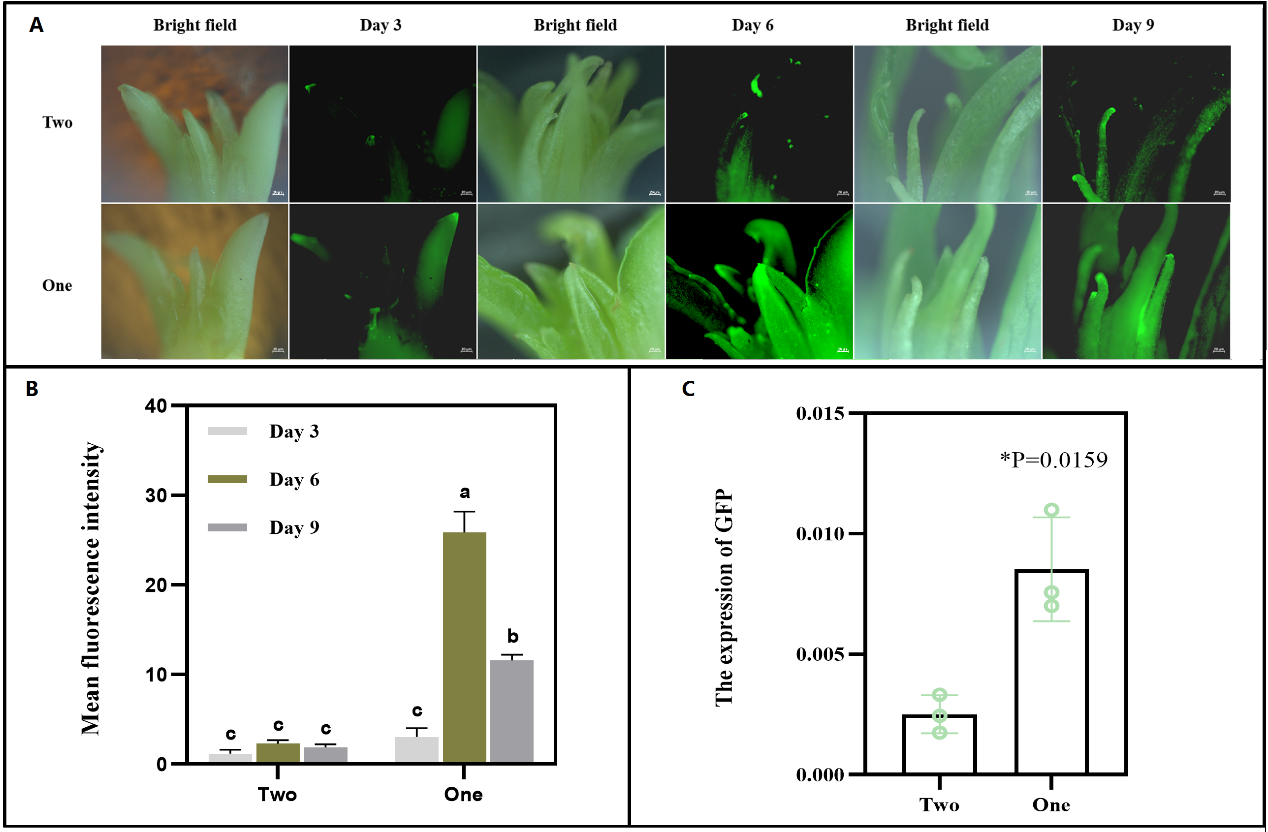  **Figure S7** Effect of cotyledon number on transformation efficiency. (A) GFP fluorescence after infection for 3, 6 and 9 days. Two= Two cotyledon, One= one cotyledon. (B) Mean fluorescence intensity. (C) Expression of *GFP* gene. Different lowercase letters indicate significant differences (*P* < 0.05) according to Duncan‘s multiple range tests by ANOVA. |
| --- |
